# Cerebral Autosomal Dominant Arteriopathy With Subcortical Infarcts and Leukoencephalopathy (CADASIL) in a 25-Year-Old Patient: A Case Report

**DOI:** 10.7759/cureus.59550

**Published:** 2024-05-02

**Authors:** Zahid Ullah, Ebad-Ur Rehman Syed, Noman Salih, Yamna Ali, Abdul Wahab, Hidayat Ullah

**Affiliations:** 1 General Medicine, Khyber Teaching Hospital, Peshawar, PAK; 2 Internal Medicine, Royal College of Surgeons in Ireland, Busaiteen, BHR; 3 Internal Medicine, Hayatabad Medical Complex Peshawar, Peshawar, PAK; 4 Medical C Unit, Hayatabad Medical Complex Peshawar, Peshawar, PAK

**Keywords:** psychiatric symptoms, cognitive decline, ischemic events, notch3 gene, hereditary, cadasil

## Abstract

Cerebral autosomal dominant arteriopathy with subcortical infarcts and leukoencephalopathy (CADASIL) is a rare genetic disorder caused by mutations in the NOTCH3 gene, resulting in subcortical infarctions and leukoencephalopathy. It predominantly affects the brain's small blood arteries, resulting in repeated ischemic episodes including transient ischemic attacks and strokes leading to cognitive impairment and mental symptoms. We provide a case study of a 25-year-old patient suspected of having meningoencephalitis. CADASIL was diagnosed based on clinical examination, imaging investigations, and genetic analysis. Optimal patient care for this complicated illness requires early detection and proper management.

## Introduction

Cerebral autosomal dominant arteriopathy with subcortical infarcts and leukoencephalopathy (CADASIL) constitutes a complex hereditary vascular disease characterized by early-onset ischemic subcortical infarctions, which are frequently accompanied by a variety of neurological insults such as migraine, cognitive impairment, mood changes, and motor deficits [1,2].

Over the years, the way we detect CADASIL has gotten better. We've made big improvements in the types of images and tests we use [3,4]. The earliest descriptions of how CADASIL appears in images from MRI scans were given in 1991. We observed hypointense and hyperintense areas within distinct cerebral regions, notably in the cortical and subcortical zones. These alterations often begin in specific areas of the brain before spreading throughout, with small pockets of damage known as lacunar infarcts which are small strokes that occur in the deep parts of the brain supplied by small arteries [5,6]. MRI images are extremely useful for determining whether somebody has CADASIL or not.

One major indicator that someone has CADASIL is if they begin to have issues before the age of 60, and we can notice alterations in their MRI images as early as age 35. This demonstrates the importance of identifying the problem early so that the patient can be counseled. Another approach to detecting if someone has CADASIL is a muscle or skin biopsy which can reveal characteristic pathological changes. Under electron microscopy, these biopsies typically show the presence of granular osmiophilic material (GOM) within the small arteries and arterioles. This test is good at determining whether someone has CADASIL, although it does not capture every instance. That is why we also do a particular type of genetic test called Notch 3 analysis, which may detect CADASIL in around 9 out of 10 instances [3]. In essence, CADASIL is a complex condition that impacts the blood vessels within the brain and generates a wide range of symptoms. We've grown better at determining who has chronic traumatic encephalopathy by capturing better brain images and running unique genetic testing. Through these diagnostic methods, we can identify CADASIL at an earlier stage, which allows for the timely initiation of supportive care strategies that may enhance the long-term quality of life for individuals with the syndrome.

## Case presentation

Our patient, a 25-year-old woman with no substantial medical history, was presented to our emergency department with several concerning symptoms. The patient presented with an acute onset of fever lasting for seven days, partially responsive to antipyretics such as paracetamol. Concurrently, she experienced a severe cephalalgia, which appeared to involve the entire cranium. While analgesics provided some relief, she did not suffer any related vomiting. In addition to these discomforts, the attendants also gave a history of a dry, nonproductive cough, a continuous irritant that exacerbated her overall discomfort.

After a comprehensive neurological assessment, we identified several concerning findings. Her Glasgow Coma Scale (GCS) score was 8 out of 15 with eye-opening to pain (+2), producing incomprehensible sounds (+2), and a withdrawal from pain (+4) indicating impaired consciousness. Her pupils were normal and reactive to light. We also discovered bilateral upgoing plantar reflexes, indicating the possibility of neurological problems. However, her deep tendon reflexes were normal, complicating her clinical presentation. Auscultation indicated bilateral chest crackles, indicating probable respiratory involvement. This discovery was consistent with her reported cough, emphasizing the necessity for a more complete approach to her care. Furthermore, neck stiffness was detected during examination, indicating possible meningeal irritation, adding to the complexity of the situation.

To better understand the underlying source of her problems, we launched a series of investigations. Fundoscopy and CT brain imaging were among the first techniques performed to rule out acute intracranial disease. To get further information, we performed a lumbar puncture, to exclude any infectious cause or any other diseases. Her CSF values, including glucose, protein, and cell count, were within normal ranges shown in Table 1; nevertheless, her erythrocyte sedimentation rate (ESR) was significantly raised at 59 mm/1st hour, above the usual range of 0-20 mm/1st hour. This discovery suggested underlying inflammation, adding another layer of complication to her situation. Additionally, the coagulation profile and viral serology tests returned normal, offering some reassurance despite the uncertainties shown in Table 2.

**Table 1:** Cerebrospinal fluid analysis WBC, white blood cells; CSF, cerebrospinal fluid

Test (CSF)	Patient's value	Reference value
Proteins (mg/dl)	35	<50
Wbcs	3	<5
Glucose (mg/dl)	78	50-75

**Table 2:** Baseline investigations aPTT, activated partial thromboplastin time; ALP, alkaline phosphatase; AST, aspartate aminotransferase; Creat, creatinine; Hb, hemoglobin; Na, sodium; MCV, mean cell volume; K, potassium; RBS, random blood sugar; WBC, white blood cells; BUN, blood urea nitrogen; PT, prothrombin time; ALT, alanine aminotransferase.

Test	Patient's value	Reference value
PT	11	<12
aPTT	27	<28
INR	1	1
Wbcs (/µl)	7200	4000 - 11000
MCV (fL)	80.5	80-100
Hb (g/dl)	12.5	11.5 - 17.5
Platelets (/µl)	170000	150000 - 450000
Alt (U/L)	35	10 - 50
Ast (U/L)	15	8 - 33
ALP (U/L)	210	< 390
Bun (mg/dl)	22	18 - 45
Bilirubin (mg/dl)	0.4	0.1 - 1
Creat (mg/dl)	0.6	0.3 - 0.9
Na (mmol/L)	137.9	135 - 150
K (mmol/L)	4.4	3.5 - 5.0
RBS (mg/dl)	115	100 - 125
ESR (mm/hour)	59	0-20

**Figure 1:**
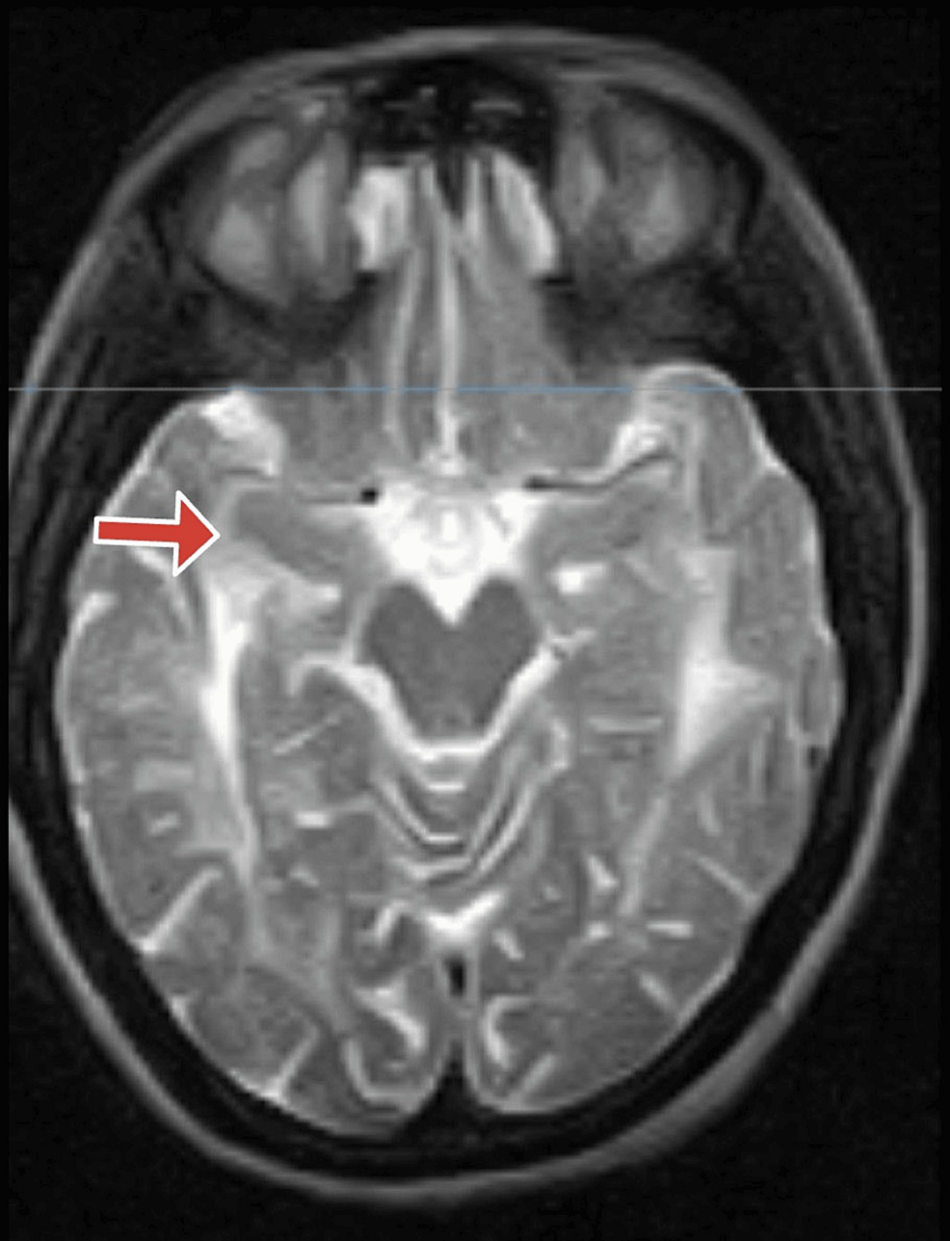
An MRI image of the brain with a red arrow showing white matter enhancement in the temporal lobe

**Figure 2:**
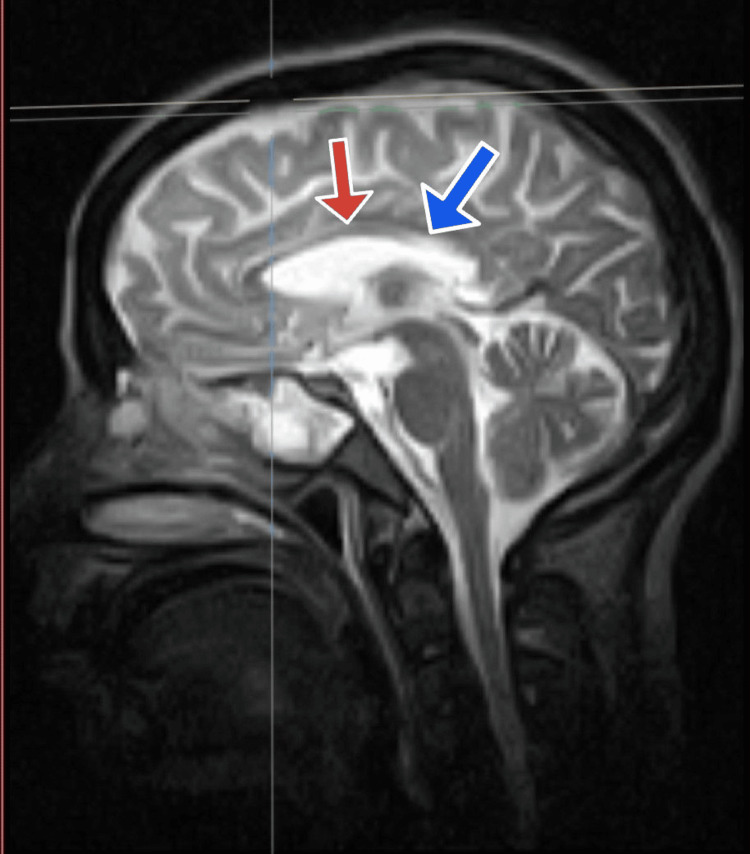
An MRI image of the brain with the red arrow shows the thinning of the corpus callosum while the blue arrow shows increased signals in the splenium

Further studies using MRI brain imaging revealed substantial findings, offering light on the possible underlying condition. Extensive alterations were seen in the white matter, notably in the parietal lobe indicating the possibility of CADASIL or other leukoencephalopathies. The imaging indicated significant increases in T2-weighted imaging (T2WI) and fluid-attenuated inversion recovery (FLAIR) signal alterations in the periventricular white matter, which extended into the subcallosal white matter, external capsule, and cerebellar hemispheres. Interestingly, subcortical u-fibers were unaffected. The anomalies were mostly concentrated in the parietal lobe, although they also affected the frontal, occipital, and temporal lobes as shown in Figure 1. The alterations were confluent, notably in the splenium, and the corpus callosum showed thinning shown in Figure 2.

Considering the clinical presentation and imaging data, we strongly suspected CADASIL, a genetic disorder that involves cerebral autosomal dominant arteriopathy, subcortical infarctions, and leukoencephalopathy. CADASIL is caused by mutations in the NOTCH3 gene, which result in vascular smooth muscle cell degeneration and consequent cerebral vascular dysfunction. However, alternative diagnoses were investigated, such as various leukoencephalopathies, vasculitis, and metabolic illnesses, emphasizing the importance of a complete diagnostic approach. To address the numerous symptoms and suspected underlying illness, a thorough treatment strategy was implemented. Level 2 analgesics (Inj tramadol 100mg as needed) were provided to treat her headaches, and antiepileptic medications (tablet levetiracetam 500mg once daily) were started owing to her seizure history. Concurrently, a tablet of clarithromycin 500mg once daily was given to treat the respiratory infection that was worsening her symptoms, and an antitussive syrup two spoons three times a day was recommended to relieve her chronic cough. Genetic testing through NOTCH3 gene sequencing was conducted, confirming the diagnosis of CADASIL and elucidating the genetic basis of the condition. This method has been shown to increase the diagnostic rate of pathogenic findings related to CADASIL. Regular follow-up sessions were organized to closely assess the therapy response and disease progression.

## Discussion

In 1993, French researchers introduced the name CADASIL to describe the condition. This hereditary condition affects tiny blood arteries in the brain, causing insufficient blood flow to specific locations, leading to various ischemia lesions and varying symptoms depending on their location. CADASIL's prevalence is unknown, although it affects roughly one in every 24,000 people, a figure that is likely overestimated. The sex ratio is 1 to 1. Symptoms appear in maturity, often around those in the age range of 30 and 40. The condition is caused by an alteration in the NOTCH3 gene located on the short arm of chromosome 19. This gene has a variety of roles throughout fetal development, most notably in the formation of artery media. As a result of a NOTCH3 gene mutation, the muscle tissue of the arteries degrades over time. The inner surface of the arteries then loses flexibility, preventing blood flow. Although arterioles in all organs can be impacted, the major effects are on the brain. Multiple infarctions develop when areas of the brain fed by these arterioles lack oxygen. Repeated minor brain infarctions lead to symptoms that worsen with time. Migraines are the most common clinical symptom of CADASIL in young people. Recurrent subcortical ischemia events can cause neurological degeneration, frontal-type dementia, and depressed psychological problems. MRI signal anomalies are always present in sick people. Abnormal brain imaging can occur before clinical signs [4,7]. MRI scans reveal hyposignal or hypersignal regions in the basal ganglia and white matter, which are commonly confluent. Hypersignals are typically symmetrical and concentrated in the periventricular and semiovale regions, indicating extensive leukoencephalopathy with lacunar infarctions of the basal ganglia and brainstem. Gradient-echo sequences may also reveal microscopic bleeding. The participation of the front poles of the temporal lobes is especially significant [8]. Distinguishing between CADASIL and leucoaraiosis on MRI can be challenging in patients with numerous infarctions along with subcortical dementia, especially in the absence of family history. These two disorders result in microangiopathic lesions. The T2 sequences reveal white matter abnormalities in both patients, primarily in the supratentorial and periventricular areas. Skin biopsy reveals vascular lesions in small arteries with thicker media due to GOM. However, skin biopsy is presently only used as a diagnostic tool in situations of persistent uncertainty after NOTCH3 gene sequencing. Mutations in this gene, which codes for a transmembrane protein, might lead to protein buildup in the media during CADASIL, perhaps causing a deficiency in the vascular wall's responsiveness. The mutation has an autosomal dominant inheritance pattern. A family examination should include an MRI, which can reveal substantial white matter abnormalities in asymptomatic patients or those with mild symptoms like headaches or depression [9]. Some closely related conditions, such as MELAS (mitochondrial encephalopathy, lactic acidosis, and stroke-like episodes), Fabry disease, and CARASIL (cerebral autosomal recessive arteriopathy with subcortical infarcts and leukoencephalopathy), were also ruled out based on clinical presentation and genetic testing. MELAS is a mitochondrial condition that often starts in childhood. It is characterized by stroke-like episodes, muscular weakness, and neurological deficits caused by mitochondrial DNA abnormalities [10]. Fabry disease is a lysosomal storage illness characterized by the accumulation of sphingolipids in multiple organs, including the kidneys, heart, and skin. It causes symptoms such as discomfort, renal malfunction, and cardiovascular problems and is inherited in an X-linked fashion [11]. CARASIL is a rare genetic condition leading to multiple strokes and dementia without cardiovascular risk factors. It involves damage to small blood vessels in the brain and is caused by mutations in the HTRA1 gene [12]. No particular therapy has been shown for CADASIL. Some publications recommend using antiplatelet medications to prevent ischemic strokes caused by atherosclerosis in individuals with ischemic brain symptoms. It is advised not to utilize anticoagulants due to the potential risk of brain hemorrhages, including symptomatic intracerebral hemorrhages and the occurrence of microbleeds in brain tissue. Additionally, it is recommended to refrain from using any vasoconstrictor treatments for managing migraine attacks during the condition because of the risk of inducing ischemia with these substances. Once the diagnosis is established, both the patient and their family must receive medical and psychological care promptly. A group for family support helps improve medical and social treatment for CADASIL sufferers [3]. Patient care must be provided in interdisciplinary and specialized settings.

## Conclusions

CADASIL syndrome, however uncommon, should be explored in young individuals with neurological symptoms. Genetic testing and close monitoring are critical for illness management. Early diagnosis enables early action and better patient outcomes. As our understanding of CADASIL grows, more research is required to optimize therapy options and improve patient outcomes. This comprehensive case report gives specific insights into the clinical course, investigations, and management of CADASIL, highlighting the significance of a multidisciplinary approach to addressing this difficult condition.

## References

[1] Joutel A, Corpechot C, Ducros A (1996). Notch3 mutations in CADASIL, a hereditary adult-onset condition causing stroke and dementia. Nature.

[2] Chabriat H, Vahedi K, Bousser MG (1995). Clinical spectrum of CADASIL: a study of 7 families. Lancet.

[3] Tournier-Lasserve E, Joutel A, Melki J (1993). Cerebral autosomal dominant arteriopathy with subcortical infarcts and leukoencephalopathy maps to chromosome 19q12. Nat Genet.

[4] Chabriat H, Levy C, Taillia H (1998). Patterns of MRI lesions in CADASIL. Neurology.

[5] Markus HS, Martin RJ, Simpson MA, Dong YB, Ali N, Crosby AH, Powell JF (2002). Diagnostic strategies in CADASIL. Neurology.

[6] Tomimoto H, Ohtani R, Wakita H, Lin JX, Miki Y, Mizuno T (2005). Distribution of ischemic leukoaraiosis in MRI: a difference from white matter lesions in CADASIL (Article in Japanese). No To Shinkei.

[7] Bousser MG, Tournier-Lasserve E (1994). Summary of the proceedings of the First International Workshop on CADASIL. Paris, May 19-21, 1993. Stroke.

[8] Tournier-Lasserve E, Iba-Zizen MT, Romero N, Bousser MG (1991). Autosomal dominant syndrome with strokelike episodes and leukoencephalopathy. Stroke.

[9] Stefanizzi S, Guichard JP, Martinez L, De Broucker T (2004). CADASIL. Rev Neurol.

[10] Momoh R, Kollamparambil S (2024). A case report of a clinically suspected diagnosis of mitochondrial encephalomyopathy, lactic acidosis, and stroke-like episodes (MELAS) syndrome with cardiac impairment. Cureus.

[11] Furia A, Ditaranto R, Biagini E (2024). Fabry disease in W162C mutation: a case report of two patients and a review of literature. BMC Neurol.

[12] Li YM, Jia W, Xin T, Fang YQ (2023). Case report: heterozygous mutation in HTRA1 causing typical cerebral autosomal recessive arteriopathy with subcortical infarcts and leukoencephalopathy. Front Genet.

